# Early stage nanocrystallization as a method of enhancement of electrical properties of lead/barium titanate doped vanadium borate glasses

**DOI:** 10.1038/s41598-023-37230-w

**Published:** 2023-08-02

**Authors:** Amany E. Harby, Ahmed E. Hannora, Atif Mossad Ali, M. M. El-Desoky

**Affiliations:** 1 Department of Physics, Faculty of Science, Suez University, Suez, Egypt; 2 Department of Science and Mathematical Engineering, Faculty of Petroleum and Mining Engineering, Suez University, Suez, 43721 Egypt; 3 Department of Physics, Faculty of Science, King Khalid University, Abha, Saudi Arabia

**Keywords:** Neuroscience, Materials science, Nanoscience and technology

## Abstract

Glass–ceramic nanocomposites (GCNs) of (10 − x) BaTiO_3_ (BT)–xPbTiO_3_ (PT)–60V_2_O_5_–30B_2_O_3_ with x = 0, 2.5, 5, 7.5 and 10 mol% were formed during heat treatment of conventional melt quenching glasses. X-ray diffraction was used to ensure glass and GCNs formation. Glasses and GCNs densities were measured by Archimedes principle. Fine polar clusters of lead titanate and/or barium titanate incorporation into vanadium borate glass matrix strongly depend on the composition. It was found out that the electrical conductivity of the initial glasses can be considerably improved by proper early stage of nanocrystallization at temperatures approaching the crystallization temperatures determined by DSC method. GCNs show massive increase in electrical conductivity (up to 6 orders of magnitude) as a function of BaTiO_3_ content. By increasing BaTiO_3_ content, the activation energy values have been found to increase. The enhancement in electrical conductivity of GCNs can be attributed to the increase in the crystalline phases in the glassy matrix which increases the concentrations of the V ion pairs.

## Introduction

Recently, glass–ceramic nano-composites (GCNs) containing ferroelectric nano-crystallites dispersed in the glass matrix received considerable interest. During the process of glass heat treatment, tiny crystals are formed inside the glass matrix, also, the level of porosity is reduced, which gives a great advantage to GCNs^1,2^. Glass and glass–ceramic systems embedded with ferroelectric materials have a remarkable applications such as radio frequency filters, actuators, flash memories etc.^3^. GCNs contaning transition metal oxide (TMO) show semiconducting behavior as a result of above one valency ions. In glass matrix contaning vanadium oxide conduction found due to small polaron hopping (SPH) between V^4+^ ↔ V^5+^. The grain size of formed nano-crystalline precepte plays significant role in enhanced electrical conductivity where hopping centers arrangment in that grains minimizing the grain boundary scattering^4–6^.

Lead titanate (PbTiO_3_) has a Peroviskite structure with good dielectric, thermal stability and ferroelectric properties. In addition, it’s high Curie temperature (490 °C) making it suitable for high temperature device applications, like transducers. The large ionic displacements of lead titanate produce large spontaneous polarization^7,8^. On the other hand, Barium titanate (BaTiO_3_) in the last few decades was highly investigated for its technological importance in electronics applications^3,9^.

For glass forming ability, vanadium pentoxide (V_2_O_5_) up to 5 mol%, acts as a network glass modifier while more than 10 mol% it acts as a network former^5^. Boron trioxide (B_2_O_3_) is a basic glass network former in borate glasses with smaller heat of fusion^10^, while BaTiO_3_ have a poor glass forming ability^3^.

Our work aims to study lead/barium titanate doped glass/GCNs prepared by conventional melt quenching technique and early stage nanocryatallization method, respectively. Moreover, the effect of barium titanate substituted by lead titanate, to produce lead free matreial, on the electrical properties was investigated. The formation of nanocrystalline phases were obtained by carful heat treatment at temperatures close to crystallization temperature.

## Experimental

Conventional melt quenching technique was used to prepare (10 − x) BaTiO_3_ (BT)–xPbTiO_3_ (PT)–60V_2_O_5_–30B_2_O_3_ with x = 0, 2.5, 5, 7.5 and 10 mol%. Extra high purity oxides reagent grade of PbTiO_3_, BaTiO_3_, B_2_O_3_ (Sigma Aldrich, 99%) and V_2_O_5_ (Fisher Scientific, 99.99%) were our starting chemicals with a total mixture of 10 g weighed in a stoichiometric ratio. After 10 min. mixing, powder of nominal compositions were placed in a platinum crucible and heated for 90 min in a Muffle furnace at 1250 °C in air. Stainless steel mold was used for casting the melt in plate shape of 2.0 mm thickness. Siemens D5000 X-ray diffractometer with nickel-filtered Cu Kα radiation was used to ensure the amorphicity of the prepared glasses under accelerating voltage of 40 kV and current of 30 mA. Shimatzou DSC 50, differential scanning calorimeter was used for thermal anaysis with heating rate of 10 °C/min under an argon atmosphere. JEOL 2100, high resolution transmission electron microscope (HRTEM) was used to confirm the presence of nano-clusters inside the glass matrix. The as-prepared glass sample was heat treated in air close to its crystallization temperature T_c_ according to DSC data at 350 °C for 2 h to obtain glass–ceramic nano-composites (GCNs). Glass and GCNs samples were coated by silver pastes for dc conductivity using Pico-ammeter type KEITHLEY 485 in temperatures range 310–450 K. At room temperature, glass and GCNS samples the average densities (ρ) were measured by Archimedes method using Toluene of density 0.866 g/cm^3^ as an immersion liquid. Density measurements were repeated five times.

## Results and discussion

**Figure 1:**
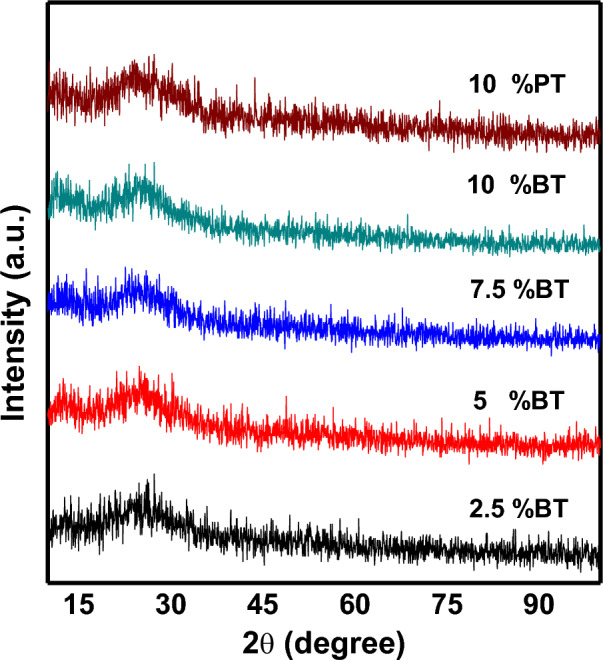
As-quenched glasses X-ray diffraction patterns.

**Figure 2:**
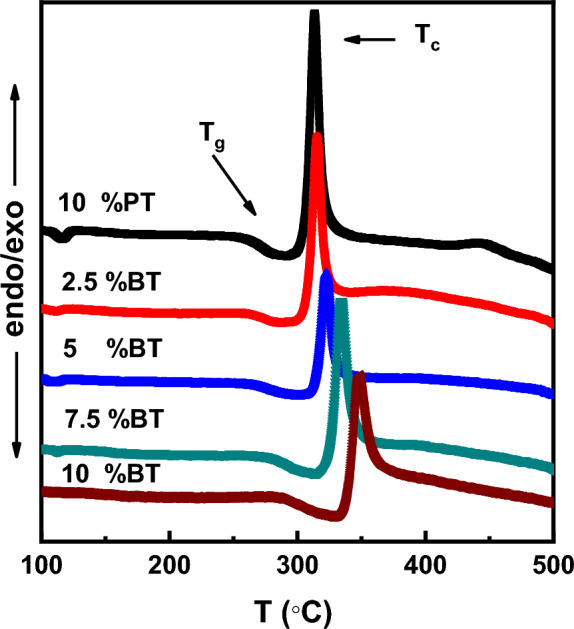
As-quenched glasses DSC curves.

XRD patterns of 10 − xBaTiO_3_ (BT)–xPbTiO_3_ (PT)–60V_2_O_5_–30B_2_O_3_ with x = 0, 2.5, 5, 7.5 and 10 mol% glass samples are shown in Fig. 1. A wide broad peak was observed at $2\uptheta \approx 25$ confirming glassy behavior of our glass system. Figure 2 shows DSC curves of the composition dependence of the glass system. Curves are characterized by an endothermic drift due to glass transition temperature (T_g_) and large exothermic peak corresponding to crystallization (T_c_). Glass transition temperature shifts to higher values with increasing BaTiO_3_ content and lies between 280 and 320 °C. Moreover, T_c_ shifted from 314 to 347 °C according to sample composition. For instance, the glass thermal stability and viscosity depends mainly on the temperature difference between the glass transition and crystallization points^11^. The temperature difference ΔT = T_c_ − T_g_ slightly varied from 36 to 31 °C with increasing BaTiO_3_ content. Also, the change in the coordination number of the network former and the construction of non-bridging oxygens (NBOs) atoms correlated with T_g_. The construction of NBOs causes a decrease into the T_g_^2,3^. In the present work, the continuous increase in T_g_ suggests continuing increase in the coordination number and destruction of NBOs atoms.

**Figure 3:**
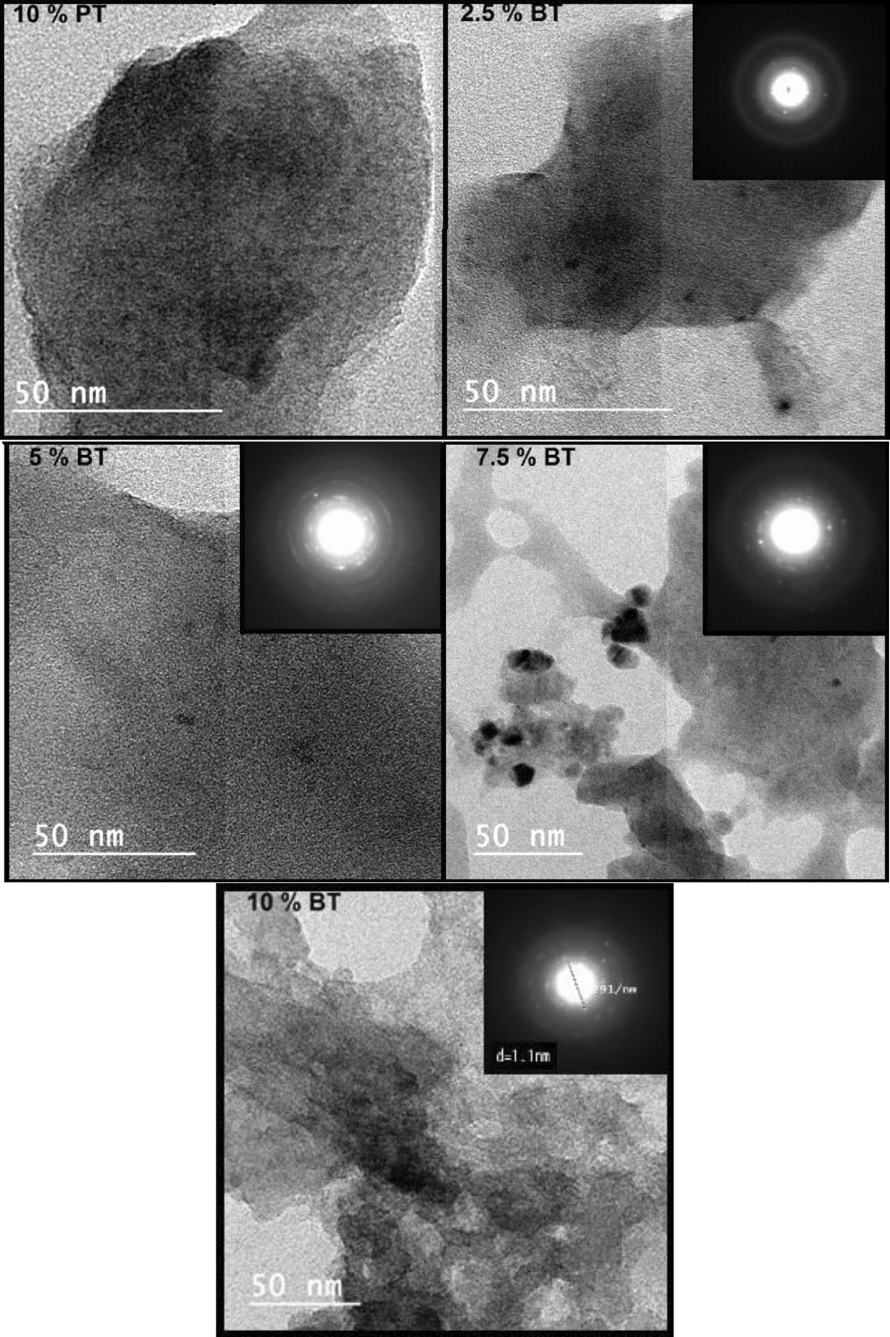
HRTEM of the as prepared glass samples the inset is the SAED.

**Figure 4:**
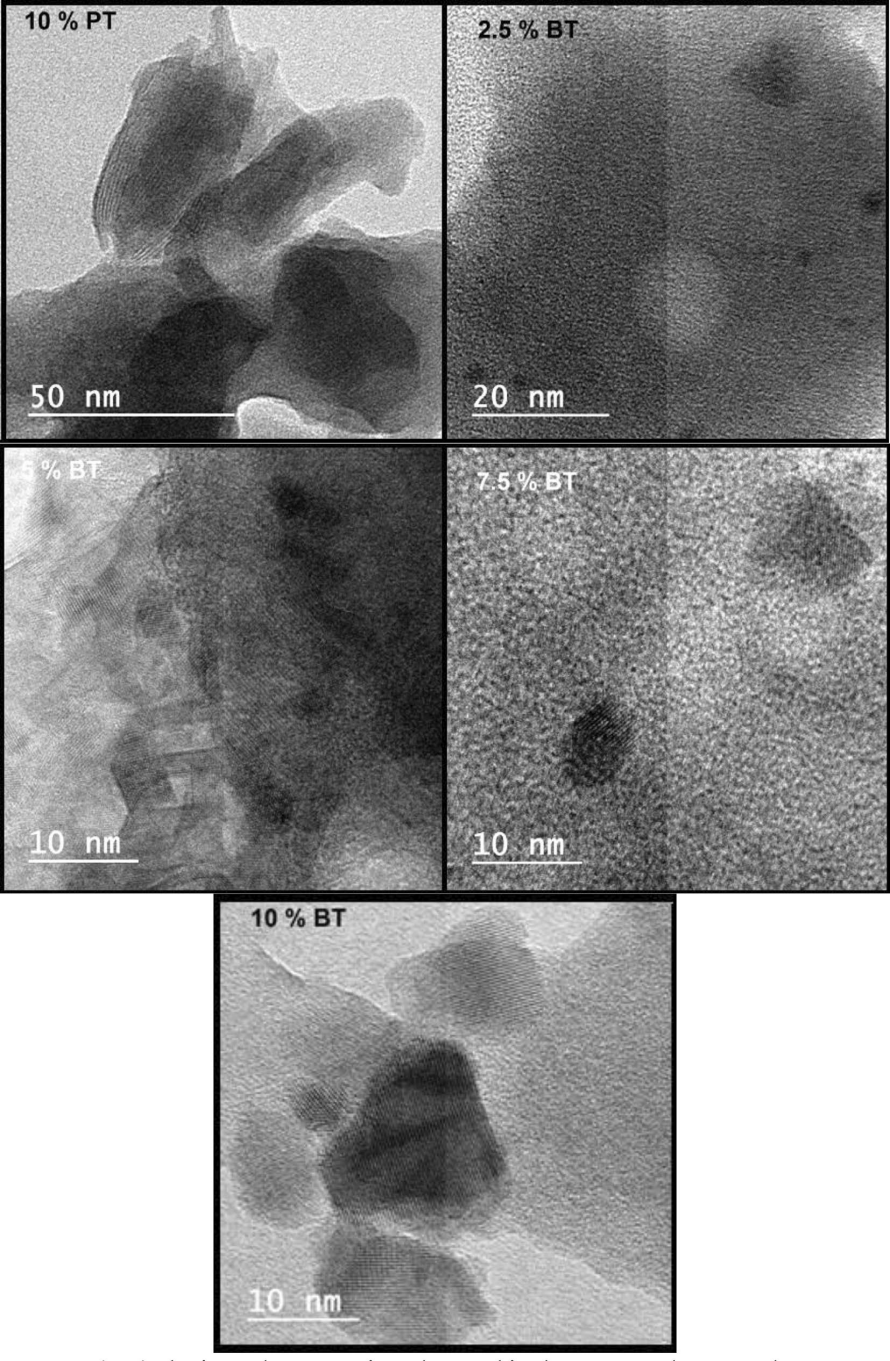
The interplanar spacing observed in the present glass samples.

HRTEM, selective area electron diffraction patterns (SAED) and the interplanar spacing of the as-quenched glass samples were presented in Figs. 3 and 4, respectively. Randomly distributed nanoclusters were precipitated in the amorphous glass matrix. The SAED patterns of the as-quenched glass samples confirm amorphous nature. However, spots in this pattern suggest the existence of nanoclusters precipitate that associated with the glass sample. Figure 4 shows the interplanar spacing of the ordered phases of the polar nano-clusters impeded in the glass matrix. The appearance of crystal defects discontinuity and presence of twin boundaries support relaxor ferroelectric-like behavior of the sample. This point was extensively studied in our recent published work^12,13^.

**Figure 5:**
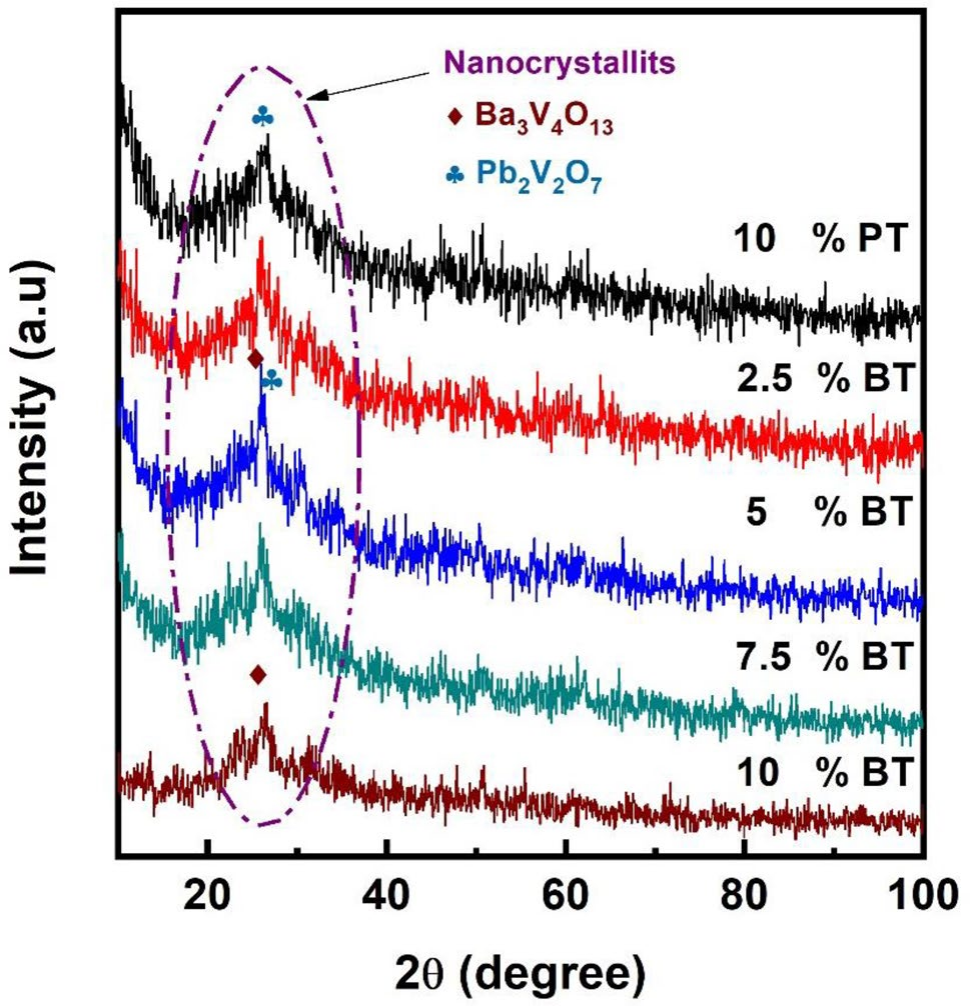
X-ray diffraction patterns of GCNs.

**Figure 6:**
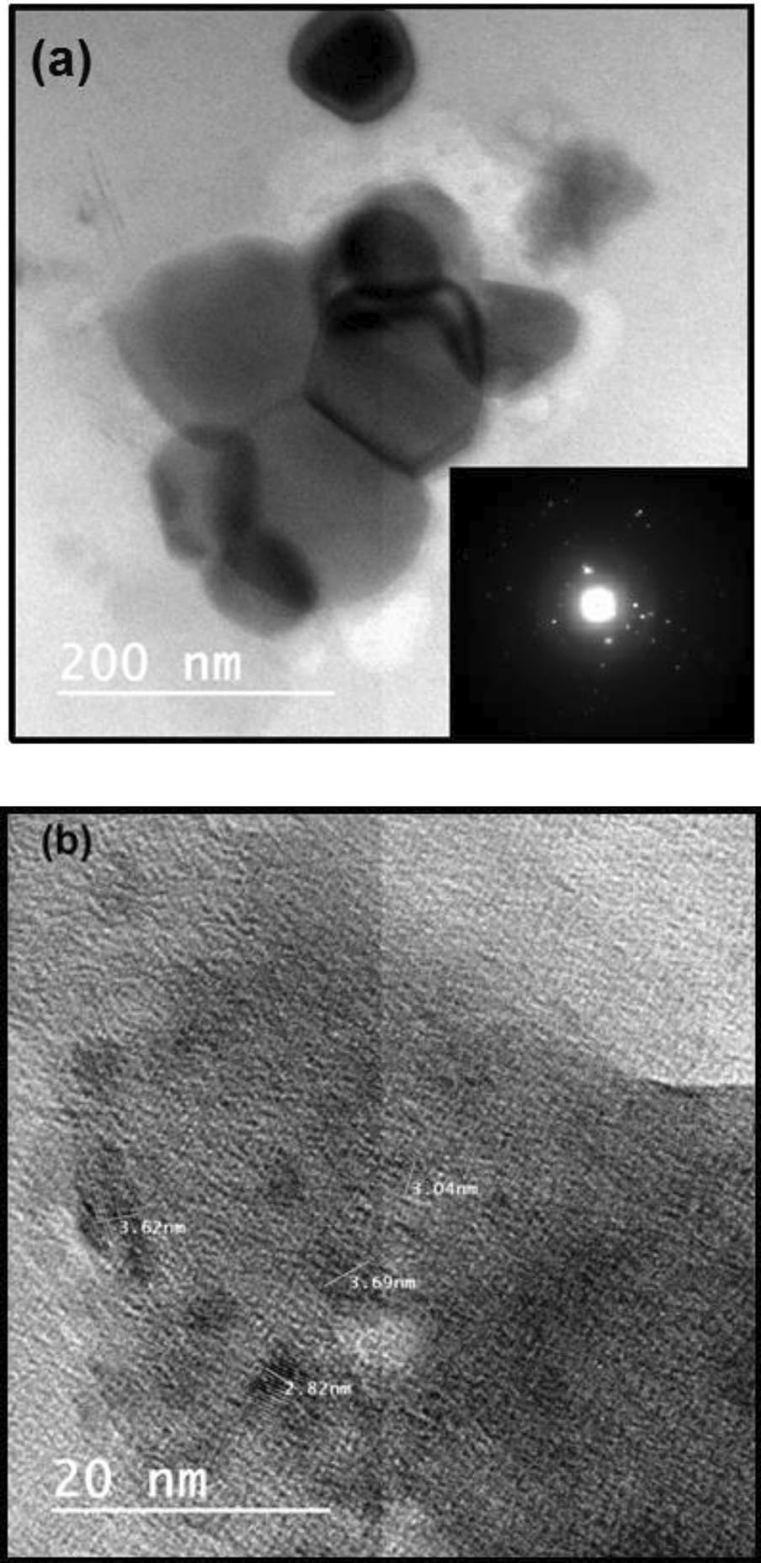
HRTEM of 5% BT glass ceramic nanocomposite (**a**) shows the presence of large particle in the glassy matrix and (**b**) shows the d spacing of a smal size grains.

Figure 5 shows XRD patterns of heat-treated glasses at 350 °C for 2 h. Broad peak still associated with partially nanocrystalized matrix confirm GCNs formation. A small (120) peak shown in the XRD result at 25.97° of 10% PT GCNs corresponds to the monoclinic Pb_2_V_2_O_7_. The XRD pattern matches with the JCPDS card no-01-084-1458. For 10% BT GCNs, the (311) peak obtained at 25.83° corresponds to monoclinic Ba_2_V_4_O_13_. Furthermore, Ba_2_V_4_O_13_/Pb_2_V_2_O_7_ coexist in GCNs with mixed PT and BT content. Scherrer equation was used to calculate the crystal size in GCNs which ranged from 7 to 15 nm^5^. The existence of a very fine crystallites embedded inside the glass matrix was observed also by using HRTEM, Fig. 6. The SAED pattern of 5%BT glass ceramic nano-composite is shown in the inset of Fig. 6. Diffraction spots from nanocrystalline phases are observed with average size larger than observed for the as prepared glass samples. It is clear from the figure that the diffraction spots show high degree of disorder.

The as-quenched glass density decreases from 3.5 up to 2.84 g/cm^3^ while molar volume increases continuously from 3.15 to 3.7 cm^3^ with the increase of the BaTiO_3_ content as shown in Table 1. After heat-treatment at 350 °C for 2 h these values increase. This increase has been attributed to the enhancement of borate tetrahedra formation besides the conversion of bridging oxygens into non-bridging oxygens. Also, the formation of noncrystalline phases compared to corresponding glasses played a significant role in higher density values.

**Table 1:** Physical properties of glasses and GCNs samples.

	BaTiO_3_ content	D (g/cm^3^)	W (eV)	Log σ (400 K)	R (nm)	N × 10^22^ (cm^-3^)	N(E_F_) × 10^21^ (eV^-1^ cm^-3^)
Glass	0	3.59	0.2881	− 7.273	0.6772	1.3484	2.457
	2.5	3.55	0.3167	− 7.357	0.6772	1.3481	2.141
	5	3.51	0.3800	− 7.844	0.6773	1.3478	3.953
	7.5	3.48	0.2871	− 8.625	0.6767	1.3513	5.625
	10	3.44	0.3232	− 9.809	0.6767	1.3510	5.624
GCNs	0	3.60	0.179	− 0.907	0.280	4.54	6.03
	2.5	3.50	0.188	− 0.999	0.282	4.42	5.59
	5	3.98	0.217	− 1.172	0.298	3.76	4.14
	7.5	3.96	0.232	− 1.174	0.299	3.74	3.84
	10	3.90	0.244	− 2.53	0.300	3.66	3.58

**Figure 7:**
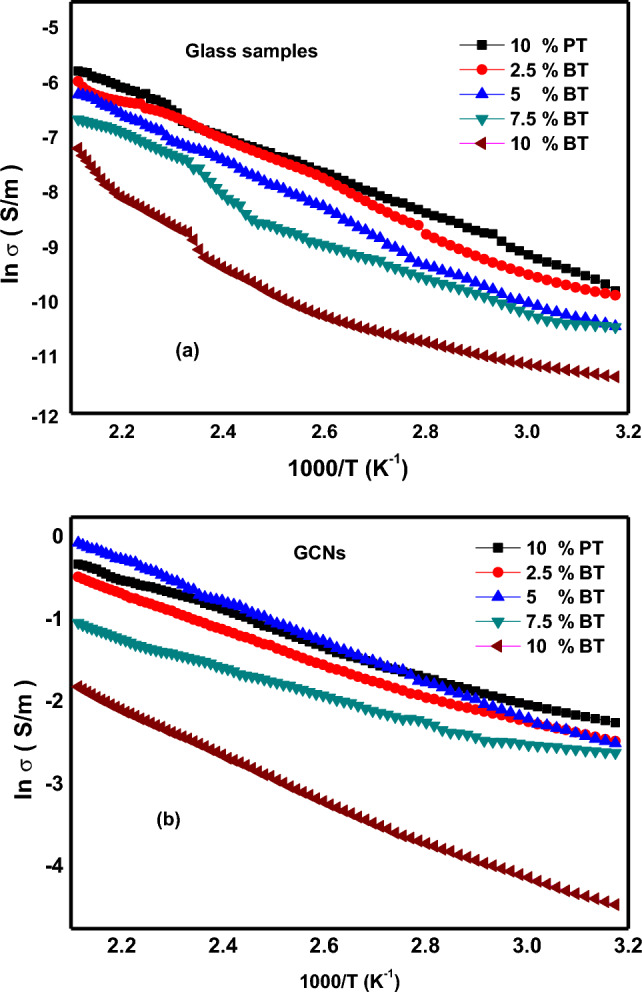
Temperature dependance of dc conductivity (ln σ) of different composition for (**a**) the glass samples (**b**) GCNs.

The electrical conduction in glasses consisting of transition metal oxides (TMOs) have been proved to be electronic in nature. The conduction process is believed to occur due to the electron hopping between the ions existing in different valence states in the glass. Figure 7a,b represents the dc conductivity (σ) variations of the glass and corresponding GCNs as a function of reciprocal absolute temperature, respectively. The figure shows a linear temperature dependence up to a certain temperature (θ_D_/2), (θ_D_: Deby temperature), and suddenly the slope changes with deviation from linearity. Along with the activation energy is a temperature dependent. Generally, for glass systems containing TMOs^14,15^, the electrical conduction at temperatures beyond θ_D_/2 is explained by the Austin-Mott theory of small polaron hopping (SPH) conduction^16,17^.

**Figure 8:**
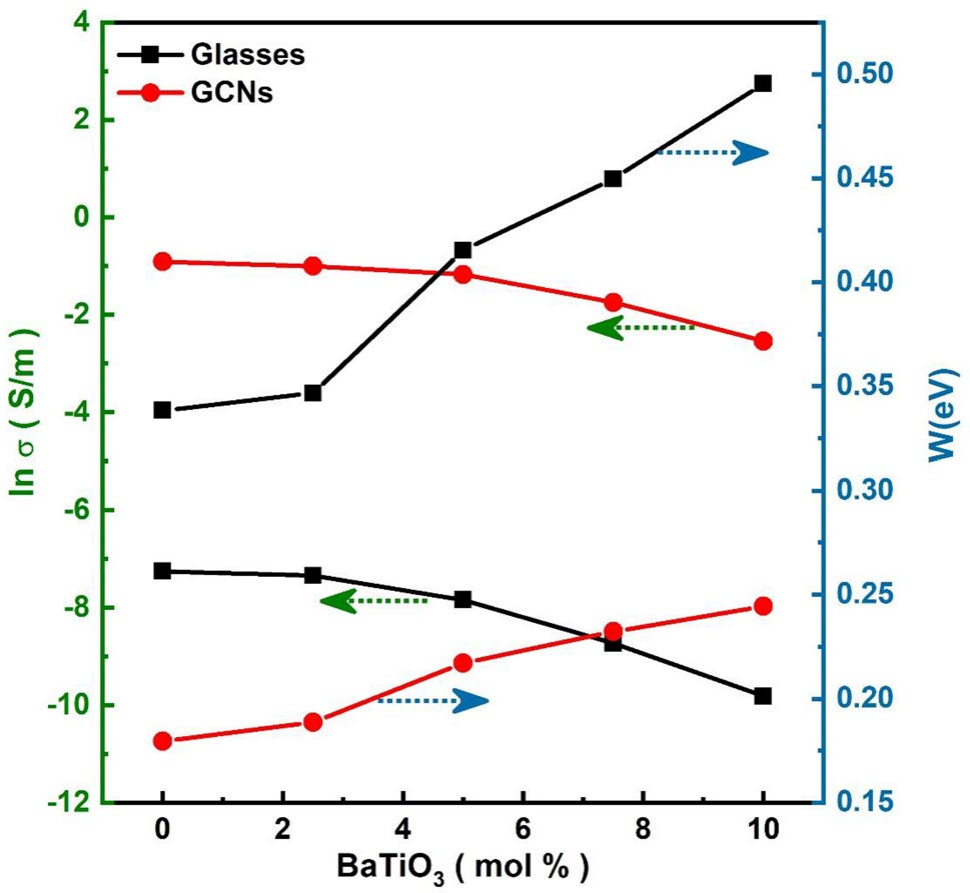
Effect of BaTiO_3_ content on activation energy (W) and dc conductivity (ln σ) at fixed temperature (400 K) for the glasses and GCNs samples.

High temperature activation energies were obtained from the slope of each curve in the highest range of the measured temperatures. The experimental conductivity data at this situation is well described by activation energy for conduction gives by:

1 $$\upsigma =\upsigma _{{\text{o}}} {\text{exp}}\left( { - {\text{W/k}}_{{\text{B}}} {\text{T}}} \right)$$

where σ_o_ is the pre-exponential factor, W is the activation energy and k_B_ is Boltzmann’s constant and T is the absolute temperature. The diversity of the high temperature conductivities and the high temperature activation energies of glasses and corresponding GCNs are shown in Fig. 8. It is clear from the figure that the conductivity decreases while the activation energy increases by increasing BaTiO_3_ content.

The activation energies for conduction of the glass samples were found to be W = 0.338–0.495 eV at high temperatures. It is known that by adding BaTiO_3_ to the glass matrix, it decreases the conductivity as a result of decreasing NBO cations^18^.This lead to decrease the open structure, through which the charge carriers can move with lower mobility. The activation energies for conduction of the GCNs were found to be = 0.179–0.244 eV. As shown from the figure, that the conductivity decreases while the activation energy increases with the increase of the BaTiO_3_ content similar as observed in the glass samples. However, there is a giant enhancement in the electrical conductivity of the GCNs. This enhancement in electrical conductivity can be attributed to the increase in the Pb_2_V_2_O_7_ nanocrystalline phases in the glassy matrix which increases the concentrations of the V ion pairs^4,5^. These results are consistent with XRD results.

**Figure 9:**
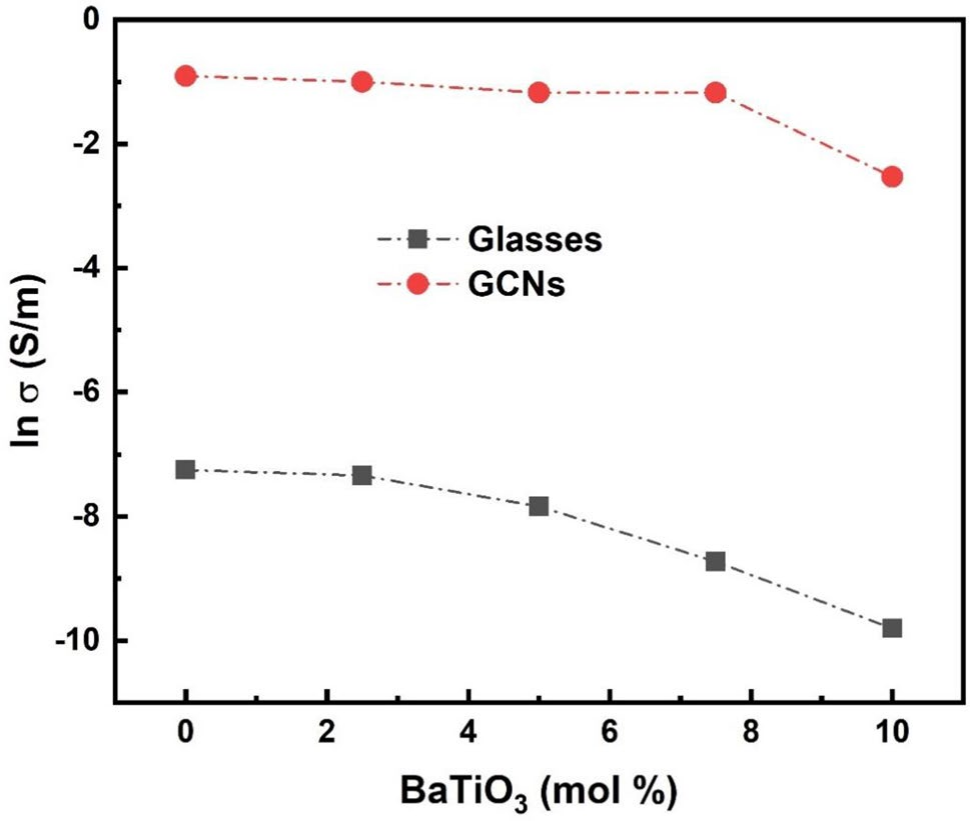
Effect of BaTiO_3_ content on dc conductivity (ln σ) at fixed temperature (400 K) for the glasses and corresponding GCNs samples.

Furthermore, the improvement of the electrical conductivity of GCNs about 6 order of magnitude under study can be explained in the following way; the key for electronic conduction in the glasses with high amount of V_2_O_5_ is the spatial distribution of V^4+^ and V^5+^ ions which are centers of hopping for electrons^19,20^. For initial glass, there is a slight random distribution of such centers. However, the early stage of nanocrystallization at temperatures near to crystallization temperature enhances crystallinity of the formation of nanocrystallites of V_2_O_5_ inside the glass matrix. Since the average size of these grains is so small, the interface region between crystalline and amorphous phases is widely ramose and strongly influences over all electrical properties of the nanomaterial. In particular, it may contain the improved concentration of V^4+^ and V^5+^ centers dispersed on the surface of V_2_O_5_ crystallites^19^. Figure 9 shows the dependence of dc conductivity (ln σ) as a function of BaTiO_3_ content for glass and corresponding GCNs at fixed temperature (400 K). It is clear from the figure that there is a massive increase in electrical conductivity of about 6 orders of magnitude after heat treatment.

**Figure 10:**
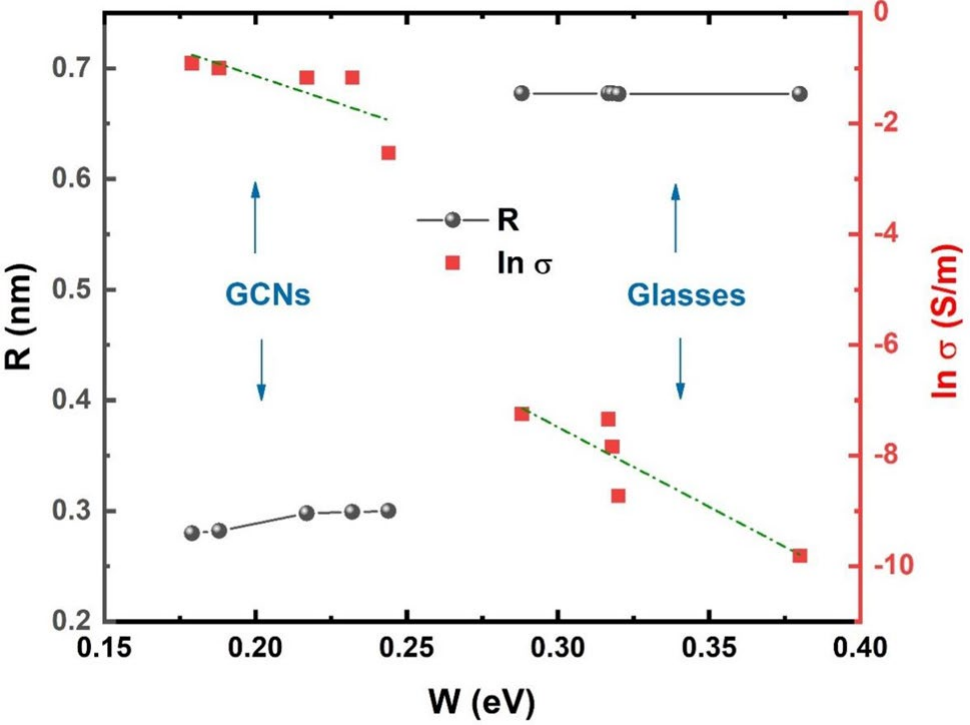
Effect of the average distance (R) on activation energy (W) and dc conductivity (ln σ) at fixed temperature (400 K) for different glasses and GCNs samples.

Moreover, the decrease in dc conductivities and the increase in activation energies of the studded samples suggest some changes in conduction mechanisms. It has been previously reported^21,22^ that in glasses which consist of vanadium, the dc conductivity is electronic and depends strongly upon the average distance, R, between the vanadium ions. The average distance, R, was calculated for the studded samples (see Tables 1 and 2) from the relation R = (1/N)^1/3^, where N is the concentration of vanadium ions per unit volume, calculated from batch composition and the measured density. The density, d, the concentration of vanadium ions per unit volume, N, and average distance, R, are shown in Tables 1 and 2 for all studded systems. The relation between the average distance R, activation energy W, and the dc conductivity, ln* σ*, for glasses and corresponding GCNs is illustrated in Fig. 10. On the other hand, the theoretical expression for that energy includes a term W = W_0_(1–r_p_/R), where W_0_ is constant and r_p_ denotes a radius of small polaron^17,21^. Overall, the electronic conductivity increases while activation energy decreases when the distance R between hopping centers decreases.

**Table 2:** Small polaron hopping parameters, hopping carrier mobility and density of state for glasses and GCNs samples.

	BaTiO_3_ content	θ_D_ (K)		ʋ_o_ × 10^13^ Hz	r_p_ (nm)	μ (cm^2^V^−1^S^−1^)	N_c_ × 10^16^ (cm^−3^)
Glass	0	670	11.70	1.39	0.2728	4.7 $\times {10}^{-4}$	9
	2.5	686	11.71	1.43	0.2729	2.2 $\times {10}^{-4}$	17
	5	714	13.46	1.48	0.2729	6.8 $\times {10}^{-4}$	3.5
	7.5	720	14.34	1.50	0.2727	1.2 $\times {10}^{-2}$	0.08
	10	754	15.15	1.57	0.2727	2.1 $\times {10}^{-3}$	0.15
GCNs	0	671	6.5	1.32	0.112	5.37 $\times {10}^{-7}$	4.6
	2.5	686	6.8	1.33	0.113	3.64 $\times {10}^{-6}$	0.6
	5	701	7.9	1.34	0.1202	4.98 $\times {10}^{-8}$	38
	7.5	722	8.5	1.31	0.1204	3.27 $\times {10}^{-7}$	3.3
	10	738	8.9	1.32	0.1213	4.68 $\times {10}^{-9}$	105

The dc conduction process in TMO glasses was investigated by Mott^16^ in a model in which the conduction process is characterized by phonon assisted hopping of small polarons between localized sates at high temperature T > θ_D_/2. For non-adiabatic hopping regime, where the electron jumping is small during each excitation the dc conductivity can be expressed by

2 $$\upsigma =\upupsilon _{{\text{o}}} {\text{Ne}}^{2} {\text{R}}^{2} {\text{C}}\left( {1 - {\text{C}}} \right)\exp \left( { - 2\upalpha {\text{R}}} \right)\exp \left( { - {\text{W/k}}_{{\text{B}}} {\text{T}}} \right)$$

The pre-exponential factor σ_o_ in Eq. (2) is given by

3 $$\upsigma _{{\text{o}}} =\upupsilon _{{\text{o}}} {\text{Ne}}^{2} {\text{R}}^{2} {\text{C}}\left( {1 - {\text{C}}} \right)\exp \left( { - 2\upalpha {\text{R}}} \right)$$

where *ʋ*_*o*_ is the optical phonon frequency which calculated from the electrical conductivity data following the relation (k_B_ θ_D_ = hʋ_o_) the values of *ʋ*_*o*_ are listed in Table 2, *N* is the transition metal ion density, *R* the average spacing between transition metal ions (= (1/N)^1/3^), *C* is the fraction of reduced transition metal ion(C = V^+4^/V_total_), W is the activation energy for hopping conduction and *α* is the tunneling factor (the ratio of the wave function decay). Suggesting a strong electron–phonon interaction, Austin and Mott showed the following^17^.

4 $${\text{W}} = {\text{W}}_{{\text{H}}} + {\text{W}}_{{\text{D}}} /2\quad {\text{for}}\;{\text{T}} >\uptheta _{{\text{D}}} /2$$

5 $${\text{W}} = {\text{W}}_{{\text{D}}} \quad \quad \quad \quad \;\;{\text{for}}\;{\text{T}} >\uptheta _{{\text{D}}} /4$$

where W_H_ is the hopping energy and W_D_ is the disorder energy which is the difference of electronic energies between two hopping sites.

6 $${\text{W}}_{{\text{D}}} = ({\text{e}}^{2} /\upvarepsilon _{{\text{o}}}\upvarepsilon _{{\text{s}}} {\text{R}}){\text{L}}$$

where ɛ_s_ is the static dielectric constant and *L* is a constant of factor 0.3.

For the adiabatic hopping regime, the electron would make transitions backwards and forwards during excitation between hopping sites. At that case αR in Eqs. (2) and (3) becomes negligible^16,17^. In this region the conductivity and the pre-exponential factor in Eqs. (2) and (3) were described as the following:

7 $$\upsigma =\upupsilon _{{\text{o}}} {\text{Ne}}^{2} {\text{R}}^{2} {\text{C}}\left( {1 - {\text{C}}} \right)\exp \left( { - {\text{W/k}}_{{\text{B}}} {\text{T}}} \right)$$

8 $${\text{and}}\;\upsigma _{{\text{o}}} =\upupsilon _{{\text{o}}} {\text{Ne}}^{2} {\text{R}}^{2} {\text{C}}\left( {1 - {\text{C}}} \right)$$

The nature of polaron hopping mechanism (adiabatic or non-adiabatic) can be acquired from a plot of ln* σ* versus activation energy W at fixed temperature T^23^. If the temperature estimated T_e_ from the slope of such a plot is close to experimental temperature T, the hopping conduction will be in the adiabatic regime. Nevertheless, if the estimated temperature T_e_ is differed than the experimental temperature T the conduction will be in the non-adiabatic regime. Figure 10 shows the relation between ln* σ* and activation energy W for glasses and GCNs at T (400 K). The estimated temperatures calculated from the slopes of the plots are T_e_ = 753.5 and T_e_ = 529.9 for glasses and GCNs, respectively which is differ than the experimental temperature. As a consequence, we can confirm the conduction mechanism in the present samples is due to non-adiabatic SPH of electrons^17^.

The non-adiabatic conduction mechanism is also confirmed from the polaron band width (J) calculation from the following relation^24^:

9 $$\mathrm{J}>{\left(\frac{2{\mathrm{kTW}}_{\mathrm{H}}}{\uppi }\right)}^{1/4}{\left(\frac{\mathrm{\hbar }{\upupsilon }_{\mathrm{o}}}{\uppi }\right)}^{1/2}\text{ (adiabatic)}$$

10 $$\mathrm{J}<{\left(\frac{2{\mathrm{kTW}}_{\mathrm{H}}}{\uppi }\right)}^{1/4}{\left(\frac{\mathrm{\hbar }{\upupsilon }_{\mathrm{o}}}{\uppi }\right)}^{1/2}\text{ (non-adiabatic)}$$

The values of J which calculated from the right hand side (R.H.S) of the Eq. (9) or (10) at fixed temperature (400 K) for the glass samples are in the range of (0.015–0.018) eV and for the GCNs are in the range of (0.003–0.014) eV. To clarify whether the polaron is in the adiabatic or in the non-adiabatic regime requires an estimate of the value of J, which can be obtained from^25^.

11 $$\mathrm{J }\approx {\mathrm{e}}^{3}{[\mathrm{N}({\mathrm{E}}_{\mathrm{F}})/({{\upvarepsilon }_{\mathrm{o}}{\upvarepsilon }_{\mathrm{p}})}^{3}]}^{1/2}$$

where

12 $$\frac{1}{{\upvarepsilon }_{\mathrm{p}}}=\frac{1}{{\upvarepsilon }_{\infty }}+\frac{1}{{\upvarepsilon }_{\mathrm{s}}}$$

where ɛ_s_ and ${\varepsilon }_{\infty }$ are the static and high frequencies dielectric constants of the samples, respectively. ${\varepsilon }_{p}$ the effective dielectric permittivity.

And $N({E}_{F})$ is the density of states at Fermi level which estimated from the following expression^16^.

13 $$\mathrm{N}\left({\mathrm{E}}_{\mathrm{F}}\right)=(3/4\uppi {\mathrm{R}}^{3}\mathrm{W})$$

Table 1 shows the values of $\mathrm{N}({\mathrm{E}}_{\mathrm{F}})$ of glass and GCNs, then, the calculated values of J given from Eq. (11) is about $\mathrm{J}\approx 0.0003$ for glasses and about $\mathrm{J}\approx 0.0001$ for GCNs which are away smaller than those calculated from the R.H.S of Eq. (10) confirming again the non-adiabatic hopping conduction at high temperature for these samples.

In the adiabatic regime the hopping conduction, W_H_, is given using J value as

14 $$\mathrm{W}-{\mathrm{W}}_{\mathrm{D}}/2\simeq {\mathrm{W}}_{\mathrm{H}}={\mathrm{W}}_{\mathrm{p}}/2={{\mathrm{W}}^{/}}_{\mathrm{p}}/2-\mathrm{J}$$

where W_p_ is the polaron binding energy, W^**/**^_p_ is the maximum polaron binding energy and W_H_ depends on R^26^.

Otherwise, for non-adiabatic hopping conduction W_H_ is given by

15 $$\mathrm{W}-{\mathrm{W}}_{\mathrm{D}}/2\simeq {\mathrm{W}}_{\mathrm{H}}={{\mathrm{W}}^{/}}_{\mathrm{p}}/2$$

By using the values of disorder energy W_D_ and activation energies W showed in Table 1, we obtain W_H_ in the range of (0.338–0.493) eV for the glass samples and in the range of (0.179–0.244) eV for the GCNs. These values are much close to W values for the studied systems.

Next, by using the values of mean spacing between vanadium ions, R (Table 1), the polaron radii can be calculated from the relation^25^.

16 $${\mathrm{r}}_{\mathrm{p}}={\left(\frac{\uppi }{6}\right)}^{1/3} \frac{\mathrm{R}}{2}$$

The values of ${r}_{p}$ and R for glass and corresponding GCNs are listed in Tables 1 and 2.

The values of small polaron coupling constant  a measure of the electron–phonon interaction, given bywere also calculated for the studied systems^16^. The estimated values of  are listed in Table 2. The value of  > 4 usually indicates a strong electron–phonon interaction^27^. The listed values show a decrease in  with decreasing BaTiO_3_ content.17

The hopping carrier mobility $\mu$ in the adiabatic and non-adiabatic hopping regions is described by the following equations^28^.

18 $$\upmu =\left(\frac{{\upupsilon }_{\mathrm{o}}{\mathrm{eR}}^{2}}{\mathrm{kT}}\right)\mathrm{exp}\left(-\frac{{\mathrm{W}}_{\mathrm{H}}}{\mathrm{kT}}\right) \, \text{(adiabatic)}$$

19 $$\upmu =\left(\frac{{\mathrm{eR}}^{2}}{\mathrm{kT\hbar }}\right){\left(\frac{\uppi }{4{\mathrm{W}}_{\mathrm{H}}\mathrm{kT}}\right)}^{1/2}{\mathrm{J}}^{2}\mathrm{exp}\left(-\frac{\mathrm{W}}{\mathrm{kT}}\right) \, \text{(non-adiabatic)}$$

Also, the carrier density (*N*_*c*_) values were calculated from the relation^29^.

20 $$\upsigma ={\mathrm{N}}_{\mathrm{c}}\mathrm{e\mu }$$

The values of $\upmu$ and N_c_ for glass and corresponding GCNs are listed in Table 2. The carrier mobility at 400 K of the studied systems is very small, suggesting that the electrons are highly localized at the V ion sites, corresponding to the strong electron–phonon interaction the large γ_*p*_^27^. As the condition of the localized for the conductive electrons is generally for $\upmu <0.01$ cm^2^V^−1^ s^−1^^27^, confirming the formation of small polaron in our samples. Also, the constant N_c_ ~ 10^18^ indicates that the conductivity of such samples is determined by hopping mobility^30–33^.

## Conclusion

Glass system of (10 − x) BaTiO_3_ (BT)–xPbTiO_3_ (PT)–60V_2_O_5_–30B_2_O_3_ with x = 0, 2.5, 5, 7.5 and 10 mol% were successfully prepared by the conventional melt-quenching technique. The amorphous nature was confirmed by XRD and DSC. HRTEM micrograph along with SAED clarified the existence of polar nanoclusters in the glass matrix and its size increases by heat treating the glass samples. The density values of the glass were found to be increase with the increase in BaTiO_3_ content while GCNs samples density values decrease. The dc conduction process is believed to occur by electron hopping between the ions existing in different valence states in the glass system (hopping will take place between the V^4+^ and V^5+^ ions). The dc conductivity in the glass system decreases while the activation energy increases with the increase of the BaTiO_3_ content. After heat treating the glass samples, there is a giant enhancement of the electrical conductivity in GCNs. The enhancement in electrical conductivity can be attributed to the increase in the nanocrystalline phases in the glassy matrix which increases the concentrations of the V ion pairs. The non-adiabatic nature of polaron hopping mechanism was confirmed in present samples. The carrier density is almost constant (*N*_c_ ~ 10^18^ cm^−3^) denoting that the conductivity of these samples is mainly determined by hopping mobility.

## Data Availability

The datasets used and/or analyzed during the current study available from the corresponding author on reasonable request.
